# 6-mercaptopurine and tofacitinib alter microbial protein expression but not composition in fecal microbiota incubations from Crohn’s disease patients

**DOI:** 10.1186/s12915-026-02569-9

**Published:** 2026-03-14

**Authors:** Heike E. F. Becker, Ronny Mohren, N. Giang Le, Luc J. J. Derijks, Daisy M. A. E. Jonkers, John Penders

**Affiliations:** 1 Division Gastroenterology-Hepatology, Department of Internal Medicine, NUTRIM Institute of Translational Research in Metabolism, Maastricht University, Maastricht, The Netherlands; 2 Department of Medical Microbiology, Infectious Diseases and Infection Prevention, NUTRIM Institute of Translational Research in Metabolism, Maastricht University, Maastricht, The Netherlands; 3 The Maastricht MultiModal Molecular Imaging (M4I) Institute, Division of Imaging Mass Spectrometry, Maastricht University, Maastricht, The Netherlands; 4 Department of Clinical Pharmacy and Pharmacology, Máxima Medical Center, Veldhoven, The Netherlands; 5 Department of Clinical Pharmacy and Toxicology, Maastricht University Medical Centre+, Maastricht, The Netherlands

**Keywords:** Crohn’s disease, Microbiome, Drugs, Microbial proteome

## Abstract

**Background:**

Crohn’s disease (CD) is a chronic, relapsing–remitting gastrointestinal inflammatory condition with a multifactorial etiology. At present, drug therapy is the most important treatment option. However, a substantial number of CD patients experience side effects and/or nonresponse to medical drugs. In part, this might be attributed to the interaction of the intestinal microbiome with xenobiotics, such as medical drugs. The aim of this study was to explore the effect of the common CD drugs budesonide, 6-mercaptopurine (6-MP), as well as tofacitinib on the CD patient’s microbiome in vitro.

**Results:**

We performed 16S rRNA gene-based bacterial community profiling and metaproteomic analyses on anaerobic ex vivo incubations of CD patient-derived fecal microbiota (FM) that were exposed to CD drugs or control conditions. Both bacterial community profiling and metaproteomics revealed larger differences in 24-h FM incubations between the five donor-derived FM samples than between the various drug incubations. Incubation of the FM of one of the donors with 6-MP or tofacitinib resulted in a significant alteration in the metaproteome when compared to the control condition, whereas no effect could be observed upon incubation with budesonide. Considering only bacterial proteins detected in at least 80% of either the drug or control FM incubations, 33 proteins were consistently more abundant and 93 less abundant in all five donor-derived samples with 6-MP incubation, distinguishing 6-MP from control conditions.

In contrast to metaproteomic analyses, bacterial community profiling only detected a significantly lower relative abundance of *Colidextribacter* in 15 µg/ml tofacitinib FM incubations. No alterations were detected in overall bacterial richness, diversity, or community structure in response to incubation with any of the drugs.

**Conclusions:**

Tofacitinib and especially 6-MP significantly affect microbial function, but barely microbial composition in vitro. These drug-induced functional changes may subsequently influence host physiology and potentially inflammation in CD. Our findings emphasize the relevance to include functional microbial studies when investigating drug–microbiota interactions. Further research is needed to elucidate the impact of 6-MP-induced microbial alterations on intestinal physiology and inflammation in CD.

**Supplementary Information:**

The online version contains supplementary material available at 10.1186/s12915-026-02569-9.

## Background

Crohn’s disease (CD) is a chronic inflammatory gastrointestinal condition with an alternating disease course [1, 2], characterized by flares and periods of remission. There is no cure for CD, and medical treatment is focused on the induction and subsequent maintenance of remission. Recommended drug classes include corticosteroids that function locally or systemically, immunosuppressants, which mainly include thiopurines, and biologicals, including anti-TNF agents [3].

Although the pathophysiology of CD is not completely elucidated, several factors are associated with disease onset. These include a genetic predisposition, environmental factors, such as cigarette smoking and low intake of dietary fiber, an aberrant immune response to the commensal microbiota, intestinal mucosal and epithelial barrier disruption, and intestinal microbial dysbiosis. In the past years, the latter factor gains increasing attention and has been shown to be related to CD onset as well as disease course [4–6]. In healthy individuals, the intestinal microbiota contributes to immune development during childhood and immune function, an intact intestinal mucosal barrier, and digestion of food [7, 8]. Although the intestinal microbiota composition varies widely between individuals, the mucosal and fecal microbiota (FM) of CD patients is characterized by a decreased diversity, decreased temporal stability, and a shift in the abundance of various bacterial taxa [9]. In addition to alterations in microbiota composition, the microbial function is also important for host health and intestinal inflammation [10].

Recent studies have demonstrated that common medical drugs can impact intestinal microbial composition in vivo as well as microbial growth in vitro. For instance, Maier et al. identified 203 human targeted drugs, which affected the growth of 40 isolated bacterial strains *in* vitro [11]. Drug-induced alterations in microbiota composition and function can potentially contribute to (impaired) mucosal healing, drug response, or drug-related side effects [12]. Also, for the various drug classes used in the treatment of CD, changes in microbiota composition or bacterial growth have been detected by in vitro, animal, and clinical studies. Although, the specific effects of these drugs on the intestinal microbiota and potentially related clinical effects are difficult to interpret due to the heterogeneity in research methodology [13]. For instance, several studies investigated the thiopurines azathioprine and 6-mercaptopurine (6-MP) on intestinal microbes, but the results were obtained from different study designs, such as in vitro bacterial strain or FM incubation, or cross-sectional in patient studies using different read-outs, including bacterial cell counting, proteomics, and 16S rRNA gene sequencing [13]. While various studies reported an effect of azathioprine or 6-MP on bacterial growth or abundance, the exact impact on bacterial species or observed changes in gut microbial composition differed between studies [13]. Combining the evidence of in vitro studies, 6-MP is found to inhibit bacterial growth of, amongst others, *Prevotella copri*, *Bacteroides fragilis*, and *Eggerthella* lenta [11, 14, 15]. In vitro mice and human studies also reported drug-microbiota interaction with other drugs that are relevant in CD treatment, including the corticosteroids budesonide and prednisolone [16–19].

Most studies have investigated the impact of medical drugs on intestinal microbiota composition, whereas the impact of drugs on microbiota function remains largely unexplored. However, compositional changes may not predict the nature of the subsequent functional changes, which can finally influence, for instance, intestinal inflammation, but may also impact microbial drug metabolism and thereby drug response and toxicity. Therefore, we aim to explore the impact of the frequently used drugs budesonide, 6-mercaptopurine, and tofacitinib on CD patient microbiota function in an ex vivo FM incubation study of five patients and to compare the extent of functional variation with the compositional changes. We chose to select these drugs because they cover three different drug classes and working mechanisms. In addition, budesonide and 6-MP are frequently used in CD treatment, and tofacitinib is a novel drug, shown to be effective in CD treatment [3, 20].

## Methods

### Study population

This study focused on the impact of medical drugs that are common in the treatment of CD [3].

Five CD patients from the Inflammatory Bowel Disease South Limburg (IBD-SL) cohort kindly provided a fresh fecal sample and completed a short questionnaire on demographics and potential confounders, including medication use and body weight (Additional file 1).

Given that budesonide, 6-MP, and tofacitinib are not recommended for administration to healthy individuals [21–23] and that the intestinal microbiome differs substantially between CD patients and healthy controls [6], potentially influencing drug–microbiota interactions, healthy subjects were not included in this study.

Prior to study participation, all participants gave written informed consent. The IBD-SL cohort was approved by the Medical Ethics Committee of the Maastricht University Medical Centre + (IBD-SL: NL31636.068.10) and registered on www.clinicaltrials.gov (NCT02130349). The study was conducted in accordance with the revised Declaration of Helsinki and in compliance with good clinical practice.

### Fecal sample collection and anaerobic fecal microbiota incubation

The participants collected complete defecations, using airtight boxes and one sachet AnaeroGen 1.5 L (Ref.: AN0025A, ThermoScientific). The sample was stored immediately at 4 °C until processing within 5 h. The sample was transferred into an anaerobic chamber with 80% Nitrogen, 10% CO_2_, and 10% H_2_ and homogenized in defined culture medium as reported by Li et al*.* with pH 7.2 [15]. The final incubations contained 2% (w/v) fecal sample, 1% dimethylsulfoxide (DMSO), and were exposed to either 55 µg/ml budesonide (Ref.: B7777, Sigma-Aldrich), 42 µg/ml 6-mercaptopurine (Ref.: 852,678, Sigma-Aldrich), 5 µg/ml (10%) or 15 µg/ml (30%) tofacitinib (Ref.: PZ0017, Pfizer®) or DMSO (control condition). We chose these concentrations based on the maximum oral intake distributed in 200 g colon content. Since 6-MP is dosed per kg body weight, we based our calculations on the approximate median body weight of CD patients, namely 70 kg [24]. All conditions were incubated in duplicate in a 2 ml 96-deep well plate covered with a PCR cover slip with ventilation holes and placed in an IKA MS 3 basic plate shaker at 500 rpm for 24 h at 37 °C in the anaerobic chamber. Thereafter, samples were processed on ice and large debris removed by three times repeated centrifugation at 300 × g for 5 min. Then, the incubation mixture was pelleted and washed with PBS at 2272 × g for 1 h at 4 °C as described by Li et al*.* [15] and subsequently stored at − 80 °C for downstream DNA and protein isolation.

### Microbiota profiling

To evaluate changes in the fecal microbial community composition following 24-h drug incubation, bacterial 16S rRNA gene amplicon sequencing was performed on both baseline fecal samples (T0) and on ex vivo FM incubations from five Crohn’s disease (CD) patients.

DNA was extracted from baseline fecal samples (T0) and washed 24-h FM incubation pellets (T24), as described elsewhere [25], and according to the adapted protocol Q from the Human Microbiome Standards [26]. The hypervariable V4 region of the 16S rRNA gene was amplified in duplicate using primer pair 515 F (5′-GTGCCAGCMGCCGCGGTAA-3′) and 806R (5′-GGACTACHVGGGTWTCTAAT-3′) containing Illumina adapters and unique barcodes as described by Caporaso et al. [27] Duplicate amplicons were pooled, purified with AMPure XP (Agencourt) according to the manufacturer’s instructions and eluted in 25 μl 1 × low TE (10 mM Tris–HCl, 0.1 mM EDTA, pH 8.0). Quantification of amplicons was subsequently performed using the Quant-iT PicoGreen dsDNA kit (Thermo Fisher Scientific, Landsmeer, the Netherlands) using a Victor3 Multilabel Counter (Perkin Elmer, Waltham, USA) and normalized to equimolar concentrations. Libraries were sequenced on an Illumina MiSeq platform (MiSeq Reagent Kit v3, 2 × 250 bp, 10% PhiX) to generate paired-end reads of 250 bases in length in both directions.

### Proteomics analysis

To explore microbial proteins and enzymatic pathways linked to drug incubation, we conducted proteomics analysis based on liquid chromatography followed by label-free quantitative mass spectrometry (LC–MS).

Proteins were isolated by diluting washed pellets in 1 ml 0.01% RapiGest SF (Ref.: 186001861, Waters) in 50 mM ammonium bicarbonate as done for DNA isolation. The supernatant was harvested after mechanical lysis and centrifugation at 16,000 x g. Isolated proteins were reduced with 20 mM Dithiothreitol (DTT) for 45 min and alkylated with 40 mM Iodoacetamide for 45 min in the dark. Alkylation was terminated by 20 mM DTT. Subsequent protein digestion was performed using Endoproteinase LysC and Trypsin (Ref.: V5071, Promega), diluted 1:25 (enzyme to protein) for 2 h at 37 °C and 750 rpm. For further overnight digestion, 50 mM ammonium bicarbonate (ABC) was added to reach a final concentration of 1 M urea, and terminated by 1% formic acid. LC–MS analysis was performed as described by Mezger et al. [28], adapted slightly using a 110 min gradient from 4 to 32% acetonitrile.

### Bioinformatic analysis and statistics

Preprocessing of 16S rRNA gene amplicon sequencing data was performed using an in-house pipeline based upon DADA2 [29] that consisted of the following steps: reads filtering, identification of sequencing errors, dereplication, inference and removal of chimeric sequences. In order to assign taxonomy, DADA2 was used to annotate down to the species level using the SILVA 138 version 2 database [30]. Decontam was used with the “either” setting, combining prevalence and frequency-based identification of contaminating Amplicon Sequence Variants (ASVs) [31]. Contaminating sequences were filtered out as well as ASVs present in less than 20% of all samples and ASVs with an overall abundance below 0.01% across samples.

Alpha diversity (observed richness and Shannon index) and beta diversity (Bray–Curtis dissimilarity) were calculated to assess within- and between-sample diversity using the online tool MicrobiomeAnalyst [32]. For statistical testing, we used the Wilcoxon signed-rank test for paired FM incubation comparisons (e.g. within-donor baseline (T0) vs. 24-h incubation (T24_control_) comparisons as well as within-donor 24-h control incubation (T24_control_) vs. 24-h drug incubation comparisons (T24_drug_)) and the Kruskal–Wallis test for between-donor differences using GraphPad Prism version 5. Beta-diversity (Bray–Curtis dissimilarity) clustering was visualized by principal coordinate analysis (PCoA) and significance evaluated by Permutational Multivariate Analysis of Variance (PERMANOVA) in MicrobiomeAnalyst [32]. Differentially abundant taxa between each drug condition and control were identified using Linear Models for Differential Abundance Analysis (LinDA) [33] using R version 4.1.2 [34].

Alpha diversity indices were visualized as data points with mean and standard deviation. Beta diversity was visualized as PCoA scores plot with two dimensions. Differences in relative abundances of bacterial taxa were visualized by a heat map in which each drug incubation was compared with the control condition.

For fecal bacterial protein annotation, the human microbiome database was used as described by Li et al. [35] and analyzed using Proteome Discoverer 2.5 (Thermofisher). For subsequent data analysis, False Discovery Rate (FDR) corrected (FDR < 1%) and normalized values were used. To explore the impact of budesonide, 6-MP, and tofacitinib (30%) on microbial protein expression, we performed microbial proteomics analysis with all experimental conditions after 24-h incubations of one randomly selected CD patient in duplicate. Subsequently, the impact of 6-MP was analyzed in all five CD patients. PCoA and hierarchical clustering analysis were conducted in Proteome Discoverer 2.5. Differential expression analyses of bacterial protein abundances were performed to detect alterations in protein expression in 6-MP FM incubations as compared to control. To limit the chance of spurious findings, solely proteins present in all donors and in at least 80% of the 6-MP exposed samples were included when examining the enrichment of proteins. When examining the depletion of proteins, only proteins that were present in all donors and at least in 80% of the control samples were included. Significance was reached at > 1.5-fold change. The PCoAs were visualized by PCoA score plots with two dimensions; hierarchical clustering analyses were visualized by heatmaps with a vertical dendrogram for protein clustering and a horizontal dendrogram for sample clustering.

KEGG orthology was applied for functional annotation using the online annotation tool GhostKOALA with option “prokaryotes” [36]. Microbial taxonomic annotation based on predicted proteins by Proteome Discoverer was conducted using the web-based application Unipept 4.0 [37]. Unipept can analyze metaproteomes based on tryptic peptides and assign those to bacterial taxa from the NCBI Taxonomy [38] using a lowest common ancestor approach [37].

## Results

### Patient population

The baseline characteristics of the CD feces donors are described in Table 1. One patient used the thiopurine pro-drug azathioprine at the time of donation, which is usually metabolized to 6-thioguanine nucleotides via 6-MP in the liver [39]. The other patients did not use any of the investigated drugs, i.e. budesonide, 6-MP, or tofacitinib.

**Table 1:** Baseline characteristics of study participants

	**CD1**	**CD2**	**CD3**	**CD4**	**CD5**
Sex (F/M)	Male	Female	Male	Male	Female
Age in years	27	45	38	43	41
BMI kg/m^2^	21.0	42.4	31.4	23.1	23.4
HBI	1	0	0	1	1
BSS	3	4	5	1	5
IBD medication	Vedolizumab	None	Vedolizumab	Adalimumab	Azathioprin, Mesalazin
Antibiotic use in past 3 months (***n***)	No	No	No	No	No
PPI use (***n***)	No	No	No	No	No
Medication change in last week (***n***)	No	No	No	No	No
Probiotic use in last week (***n***)	No	No	No	No	No
Alternative diet (***n***)	No	No	No	No	No
Current smoker (***n***)	No	No	Yes	No	No
Current pregnancy (***n***)	No	No	No	No	No

*BMI* body mass index, *HBI* Harvey Bredshaw Index, *BSS* Bristol Stool Scale, *PPI* proton pump inhibitor

### The impact of 24-h FM incubations on microbiota composition

To assess whether the 24-h FM incubation procedure itself altered the microbial composition, we compared each baseline (T0) fecal microbiota (FM) sample with its corresponding 24-h incubated FM sample without drug incubation (T24_control_) using 16S rRNA gene amplicon sequencing.

Across donors, the median change in observed richness after 24 h of incubation was + 3 ASVs (range, –8 to 13), indicating only minor shifts in the number of detected taxa. In comparison, the differences in richness between donors at baseline were larger (median 8 ASVs; range, –80 to 92). Similarly, bacterial diversity, as measured by the Shannon index, showed a median change of –0.373 (range, –0.174 to –0.415) within samples after incubation, whereas baseline differences between donors were greater (median 0.241; range, –0.841 to 1.163).

**Fig. 1:**
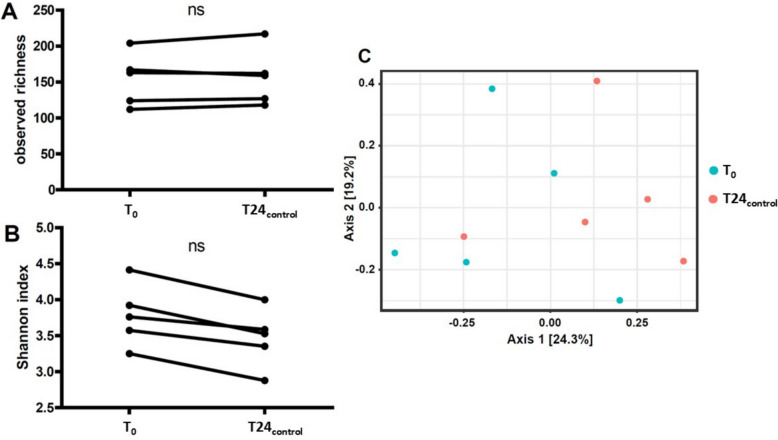
The effect of ex vivo incubation on microbiota diversity. The observed richness (**A**) and the Shannon index (**B**) were not significantly different before (T0) and after 24-h (T24_control_) incubation of fecal microbiota (FM) of CD patients (Wilcoxon signed-rank test *p* = 0.625; *p* = 0.063, respectively). **C** The Bray–Curtis index was not significantly different between the baseline fecal samples (T0) and the respective 24-h FM incubations (T24_control_) (PERMANOVA, *p* = 0.152)

Statistical testing confirmed that neither observed richness (Wilcoxon signed-rank test, *p* = 0.625; Fig. 1A) nor Shannon diversity (*p* = 0.063; Fig. 1B) differed significantly between baseline (T0) and 24-h post-incubation (T24_control_) samples. Likewise, overall community structure assessed by the Bray–Curtis dissimilarity index showed no significant change (PERMANOVA *p* = 0.152; Fig. 1C).

Together, these findings indicate that 24-h ex vivo incubation without drugs did not substantially affect the composition or diversity of the microbiota, and therefore is unlikely to bias subsequent analyses. All downstream comparisons were therefore performed using the 24-h incubated FM samples.

### The impact of 24-h drug incubation on fecal microbiota composition

To evaluate whether incubation with different IBD drugs affected the microbiota composition, we profiled the 24-h FM incubations from five CD patients under each experimental condition.

**Fig. 2:**
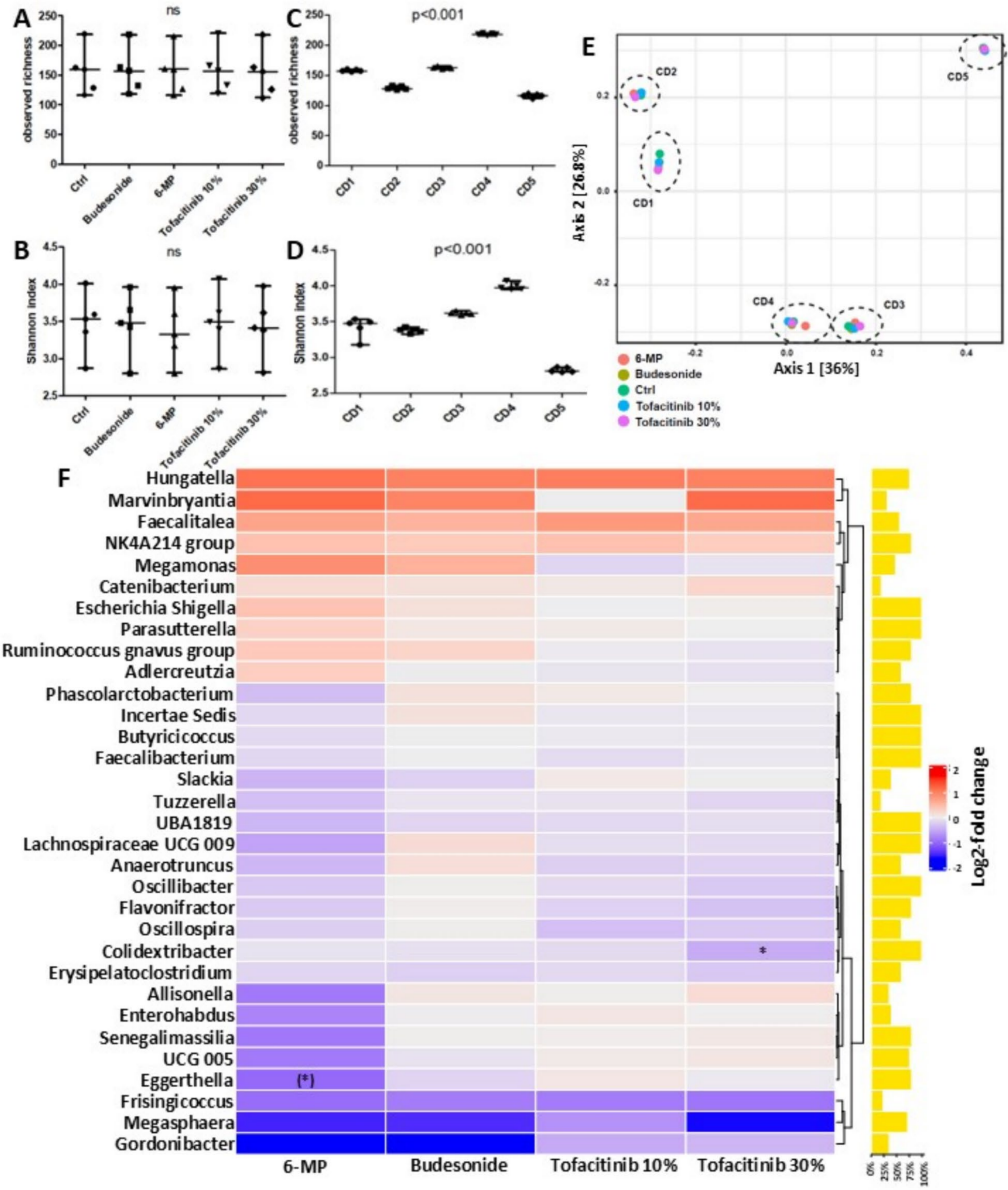
Alterations in microbial composition upon incubation with budesonide, 6-mercaptopurine, or tofacitinib. Observed richness (**A**) and Shannon index (**B**) between experimental conditions (none significant, see Table 2) and between donors (both *p* < 0.001; **C**, **D**, respectively). Principal coordinate analyses on Bray–Curtis dissimilarity (**E**). Experimental conditions, indicated by the different colors, did not result in significant clustering of samples (*p* = 1.0), whereas samples from the same donor, highlighted by the dashed ellipses, did significantly cluster together (*p* = 0.001). Heatmap of log2-fold changes in relative abundances of bacterial genera upon drug incubation as compared to the control condition (**F**). The prevalence of the genera is depicted as vertical bars. LinDA *p* < 0.05; () = *p* < 0.1

When comparing, within each donor, the drug-exposed incubations (T24_drug_) to their respective control incubation (T24_control_), no significant differences were observed in either bacterial richness (Fig. 2A) or Shannon diversity (Fig. 2B) (Wilcoxon signed-rank test *p* > 0.05, GraphPad Prism v5; Table 2). Similarly, overall community structure assessed by the Bray–Curtis dissimilarity index did not differ significantly between control and drug-treated incubations (PERMANOVA *p* = 1.0; Fig. 2E).

**Table 2:** Comparison of microbial richness and diversity between the experimental conditions

Comparison	Observed richness *p*-value	Shannon index *p*-value
T24_control_ vs T24_Budesonide_	0.586	1.000
T24_control_ vs T24_6-MP_ 6-MP	0.345	0.125
T24_control_ vs T24_Tofacitinib 10%_	0.188	0.625
T24_control_ vs T24_Tofacitinib 30%_	0.136	0.313

In contrast, when comparing microbiota profiles across donors—combining all experimental incubation conditions—there were significant differences in both observed richness and Shannon diversity (Kruskal–Wallis test, both *p* < 0.001; Fig. 2C–D) as well as in community composition (Bray–Curtis PERMANOVA *p* = 0.001; Fig. 2E).

At the genus level, few taxa showed consistent changes across donors. Differential abundance analysis revealed that *Colidextribacter* was significantly decreased after incubation with tofacitinib (–30%, *p* < 0.05), and *Eggerthella* tended to decrease in incubations exposed to 6-MP (*p* < 0.1; Fig. 2F).

Together, these results indicate that inter-individual differences between donors outweighed the modest, drug-specific effects on microbial diversity and composition under the experimental conditions tested.

### The impact of 24-h drug incubation on the microbial proteome in FM incubations from a single CD patient

**Fig. 3:**
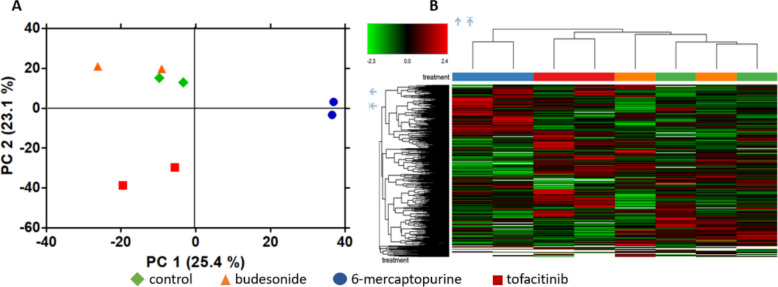
Proteome-based differences between drugs within fecal microbiota incubations of one CD patient. Principal component analysis (**A**) and heatmap (**B**) comparing the impact of budesonide, 6-mercaptopurine, tofacitinib, and control on the fecal microbial protein expression of one CD patient after 24 h incubation in duplicate

To explore the impact of budesonide, 6-MP, and tofacitinib (30%) on microbial protein expression, we performed microbial proteomics analysis with all experimental conditions of one randomly selected CD patient in duplicate. Principal component analysis showed a clear distinction between 6-MP and control (*i.e.* no drug added), and between tofacitinib and control, whereas budesonide could not be discriminated from the control condition (Fig. 3A). Hierarchical clustering analysis identified the metaproteome of the 6-MP-treated sample as being the most distinct from the control condition (Fig. 3B). Therefore, we decided to further explore the impact of 6-MP on microbial protein expression for all five CD patients.

### The impact of 6-MP incubation on the microbial proteome in 24-h FM incubations from five CD patients

**Fig. 4:**
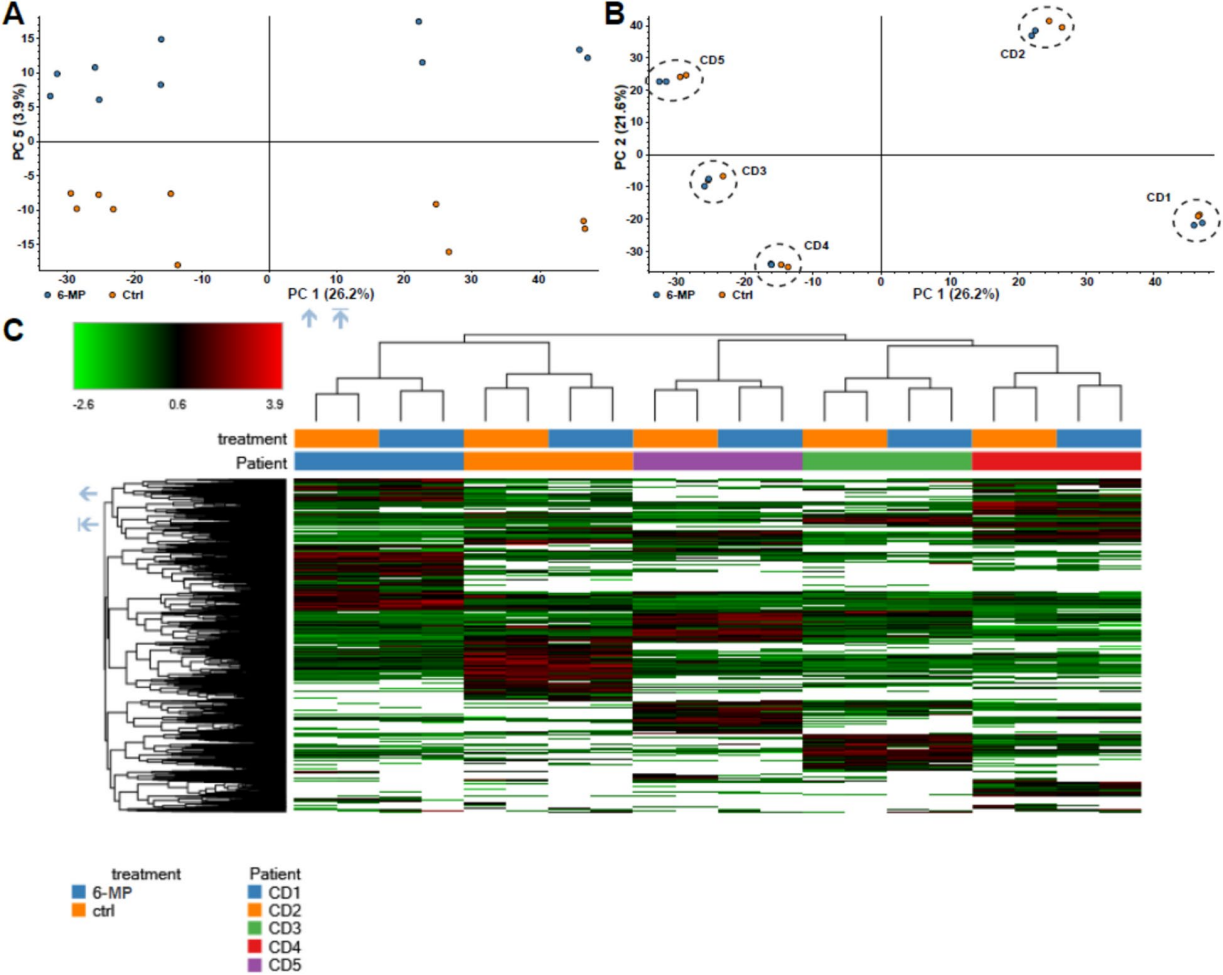
Metaproteome-based differences between 6-mercaptopurine incubation and control comparing five CD patients. Principal component analysis discrimination based on 6-MP incubation in PC 1 and PC 5 (**A**) and based on donor in PC 1 and PC 2 (**B**). Heatmap of protein abundances (**C**) comparing the effect of 6-MP and control on the FM incubations

Principal component analysis revealed a clear distinction based on 6-MP incubation by PC5 (Fig. 4A), while inter-individual differences between donors had the largest impact on variations in the metaproteome (Fig. 4B). Hierarchical clustering analysis confirmed a higher discrimination based on the donors, though with consistent discrimination based on 6-MP incubation for each patient’s FM incubation (Fig. 4C).

**Fig. 5:**
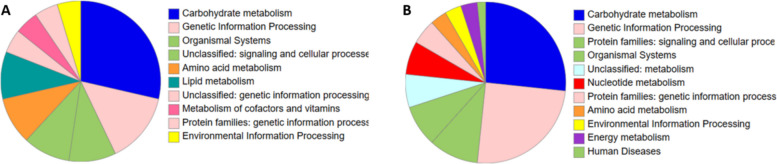
Functional annotation of 6-MP associated proteins. Pie charts displaying microbial proteins that were increased (**A**) or decreased (**B**) due to 6-MP incubation. Proteins were annotated using KEGG orthology assignment with the GhostKOALA [36] online tool

To further investigate altered biological processes and to identify bacterial proteins that are increased or decreased due to 6-MP incubation, we performed differential expression analyses of protein abundance. After 6-MP incubation, 33 proteins were significantly increased (> 1.5-fold change) and 93 proteins were significantly decreased of the 4178 proteins that were initially identified by Proteome Discover from all samples in total. Applying KEGG orthology assignments, 63.6% of the increased proteins could be annotated (Fig. 5A, Additional file 2: table S1). In general, several proteins were annotated to different pathways. Most of these were found in pathways related to carbohydrate metabolism (six proteins), covering different physiological processes, including glycolysis, citrate cycle, and pyruvate metabolism. The second most common proteins were annotated to the ribosome (three proteins).

Of the decreased proteins, 64.5% could be annotated. Most of the decreased proteins were also found to be part of carbohydrate metabolism (16 proteins), mainly including glycolysis, citrate cycle, and pyruvate metabolism (Fig. 5B, Additional file 3: table S2). The second most decreased proteins were related to genetic information processing (15 proteins), most of them to the ribosome (eight proteins). Besides the ribosome, seven proteins were annotated to amino acid metabolism, two to translation factors, and one as aminoacyl-tRNA biosynthesis, which together are all involved in protein synthesis. Further, three proteins were related to purine metabolism and two to pyrimidine metabolism, which is both related to DNA synthesis and bacterial growth. Moreover, three proteins are related to transporters, of which one was annotated as trimeric autotransporter adhesin, which is related to bacterial adhesion [40].

### Microbial taxonomic annotation of 6-MP associated proteins

Proteins that consistently increased or decreased in abundance (in ≥ 80% of samples) following 6-MP incubation were taxonomically annotated using Unipept 4.0 [37]. For this analysis, we used the predicted protein sequences generated by Proteome Discoverer, as direct submission of peptide sequences resulted in low annotation coverage.

**Fig. 6:**
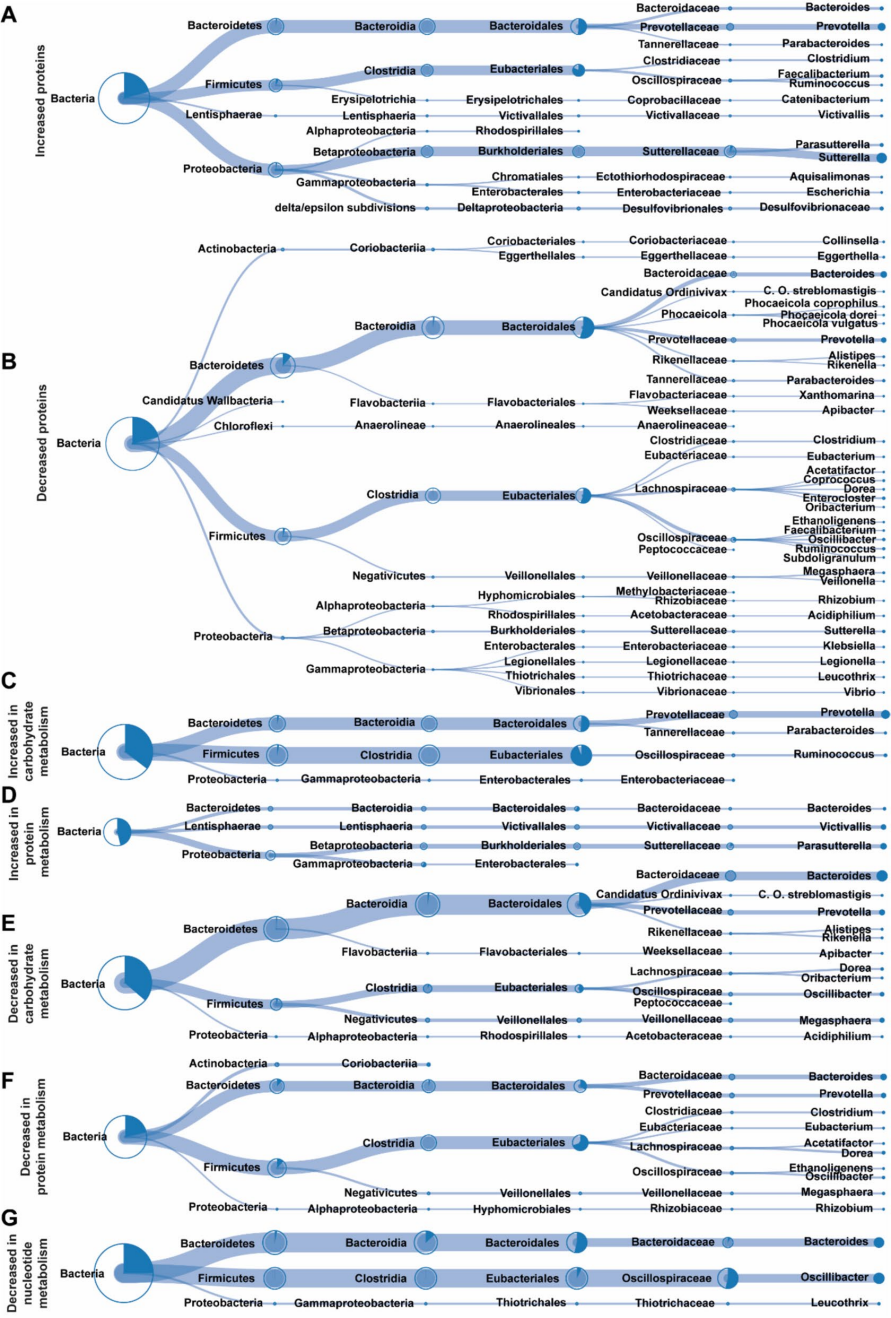
Taxonomic annotation of peptides associated with 6-mercaptopurine (6-MP) incubation. Bacterial taxonomic annotation of proteins that were increased (**A**) or decreased (**B**) in all donors due to 6-MP incubation. Bacterial taxonomic annotation of proteins that were increased (**C**, **D**) or decreased (**E**, **F**, **G**) due to 6-MP incubation and related to carbohydrate metabolism (**C**, **E**), protein synthesis (**D**, **F**), or nucleotide metabolism (**G**). Bacterial taxa are represented up to genus level and the integrated pie charts and line thickness indicate the fraction of the respective lower taxa that could be annotated. Figures were extracted from Unipept 4.0 [37] and adapted for readability

Taxa associated with proteins increased after 6-MP incubation were primarily assigned to the phyla Bacteroidetes (predominantly *Prevotella*), Firmicutes, and Proteobacteria (notably Sutterellaceae), and occurred at broadly comparable relative abundances (Fig. 6A). In contrast, proteins that decreased were mainly derived from Bacteroidetes (especially *Bacteroides*) and, to a lesser extent, from Firmicutes (Fig. 6B).

When considering functional categories, increased proteins related to carbohydrate metabolism were mostly derived from Firmicutes (particularly Eubacteriales), followed by Bacteroidetes (*Prevotella*) and a small contribution from Proteobacteria (Fig. 6C). Conversely, carbohydrate-metabolism proteins that decreased after 6-MP incubation were mainly associated with Bacteroidetes (*Bacteroides* and, to a lesser extent, *Prevotella*), and secondarily with Firmicutes (Eubacteriales) (Fig. 6E).

Proteins involved in protein synthesis—predominantly ribosomal proteins—that increased after 6-MP incubation were mostly derived from Proteobacteria (*Parasutterella*) (Fig. 6D). In contrast, decreased protein-synthesis-related proteins were largely affiliated with Firmicutes (Eubacteriales) and Bacteroidetes, with *Bacteroides* slightly outnumbering *Prevotella* (Fig. 6F).

Finally, a small subset of four decreased proteins associated with nucleotide metabolism originated from Bacteroidetes (*Bacteroides*), Firmicutes (Oscillospiraceae), and, to a lesser extent, Proteobacteria (*Leucothrix*) (Fig. 6G).

## Discussion

Recent studies showed that numerous medical drugs can influence the growth of various intestinal bacterial strains [11]. In CD, drug-induced changes in microbiota composition and function may contribute to the reduction of mucosal inflammation or affect drug response [12]. Therefore, this study aimed to explore the effect of three CD drugs, namely budesonide, 6-MP, and tofacitinib, on CD patient microbiota composition and function using an ex vivo FM culture system.

In the present study, we found no significant effect of the selected CD drugs on the overall microbial community structure. However, when exploring the effects of these drugs on the fecal metaproteome for a single patient, profound effects were found after both 6-MP and tofacitinib incubation. Furthermore, extending the analyses of 6-MP incubation to fecal samples of all five CD patients led to over- and underrepresentation of specific proteins in all donors.

In previous years, numerous studies have investigated the intestinal microbiota composition based on 16S rRNA marker gene sequencing. Thereby, an altered microbiota composition was found to be associated with CD onset and disease course as well as with treatment response [5, 9, 41]. When CD drugs can beneficially alter the microbiota composition resembling a “healthier” microbiota, this might add to the treatment response of these drugs in addition to their known direct immunomodulatory properties. For this study, we chose budesonide, 6-MP, and tofacitinib, because the former two are frequently used for CD treatment and have different working mechanisms, while tofacitinib is a new oral drug that has been found effective for CD treatment [3, 20]. For all drugs, we chose (supra-)physiological concentrations that may be found in the terminal ileum and colon. To study both FM community profiling and metaproteomics, we established a small size ex vivo FM incubation system based on the protocol by Li et al*.* [15]. After 24-h incubation, the microbiota composition remained stable, without major bacterial loss or overgrowth due to the experimental microenvironment. While this indicates that the microbial community structure was preserved, model-specific alterations in the bacterial metaproteome over the 24-h ex vivo incubation period were not assessed. Therefore, potential time-dependent proteomic changes independent of drug incubation cannot be excluded. Nevertheless, all experiments included donor-specific control incubations without drug treatment, allowing reliable comparison of drug-exposed samples to their matched controls.

Based on previous cross-sectional clinical or in vitro strain culture studies, we expected to detect compositional changes induced by budesonide and 6-MP [11, 14, 16], while tofacitinib has, to our knowledge, not yet been investigated. Interestingly, we could not detect any significant alterations in microbial richness, diversity, and community structure. Only the *Colidextribacter* genus was significantly less abundant in FM incubations after 24-h incubation with 30% tofacitinib. While not statistically significant, *Eggerthella* tended to decrease upon 6-MP exposure in line with a previous proteomics-based human ex vivo fecal incubation study [15]. A potential explanation for the almost complete absence of compositional changes may include that the microbial community within an FM incubation is more resilient to disturbances by xenobiotics, such as medical drugs, when compared to in vitro culture of single bacterial strains [42], at least upon a single dose of a certain drug. In addition, the large inter-individual variations in microbiota composition and the small number of samples might also limit the identification of consistent shifts across individuals in response to drug incubation. Moreover, the incubation period of 24 h might be too short to detect shifts in the microbiota composition, especially since free DNA and DNA from nonviable bacterial cells will also be picked up in the bacterial community profiling analysis [43].

Based on previous studies, we initially expected alterations in the microbiota composition in response to the examined drugs. Together, two in vitro studies reported growth inhibition due to 6-MP of several bacterial strains, including *Prevotella corpi*, *Campylobacter concisus*, and *B. *fragilis [11, 14]. To explore general bactericidal effects, future ex vivo FM incubation studies should include analyses on absolute bacterial abundances in response to incubation with IBD drugs and confirm potential findings in more complex ex vivo models or clinical intervention studies.

Recently, research corroborated that the microbiota composition itself may not be the dominant driver of microbiome–host interactions. Instead, functional readouts, such as the microbial proteome, may indicate more directly the impact of microbial changes on host physiology [10]. Therefore, for the first time, we analyzed the bacterial metaproteome in response to our selected CD drugs. When first screening each drug in one patient-derived FM incubation, the metaproteome could discriminate 6-MP and tofacitinib, but not budesonide, from the control condition. Although 6-MP and tofacitinib did not significantly change the microbiota composition, the metaproteomic analyses clearly showed that both have a significant impact on feces-derived bacterial proteins.

Since 6-MP appeared to have the largest impact on the metaproteome, we chose to explore its impact in all five CD patient-derived FM incubations. Intriguingly, besides an individual microbiota composition, which has often been described in previous research [9, 44], the bacterial metaproteome also appeared to be highly individual. This finding is in line with previous metaproteomic research on ex vivo FM incubation of healthy volunteers, investigating other drug compounds than the present study, which showed that regardless of drug incubations, the proteomes of the donors cluster together in a PCoA [15]. Despite the highly individual metaproteomes, 6-MP and tofacitinib appeared to induce drug-specific alterations to the metaproteomes. The effect of 6-MP appeared more pronounced, which is consistent with current knowledge of its pharmacological mechanism and known microbe–drug interactions.

Previous in vitro studies have demonstrated that several commensal intestinal bacteria, including *B. fragilis* and *Escherichia coli*, can metabolize 6-MP into its pharmacologically active derivatives 6-thioguanine nucleotides (6-TGN), through a series of three catalytic reactions [14]. This bacterial conversion may contribute to the stronger metaproteomic response observed following 6-MP incubation. Furthermore, in humans, 6-TGN can interact with and inhibit guanosine triphosphatase (GTPase) on CD28-positive immune cells [39]. Because numerous GTPases are conserved among bacterial taxa, where they regulate essential processes, such as translation [45], it is plausible that 6-TGN may also interact with bacterial GTPases, potentially influencing microbial cellular functions. In contrast, to our knowledge, tofacitinib has yet to be shown to interact with microbes. When considering its working mechanism, tofacitinib is a selective inhibitor of Janus kinase (JAK) 1 and 3 by reversibly binding to its ATP binding site [46]. JAK molecules are not present in bacteria or other microbes [47, 48]; however, ATP binding sites of microbial proteins could serve as targets for tofacitinib. Further mechanistic investigations are requested to identify potential microbial targets for tofacitinib.

When extrapolating the impact of 6-MP on the FM of all five patients, significant and consistent over- and underrepresentation of protein clusters could be detected in the FM incubations of all donors. The largest shifts have been annotated to bacterial carbohydrate metabolism. The affected taxa may either have de- or increased their carbohydrate metabolism in general or altered related pathways. For instance, the mannose phosphotransferase system component EIIAB, which can phosphorylate D-mannose among other sugars [49], is increased due to 6-MP incubation. This could indicate an increased production of D-Mannose-6P. Since the other enzymes, which can conduct the same function (*i.e.* hexokinase and mannokinase) [50, 51] were not found to be decreased, we can assume that D-Mannose phosphorylation may indeed be increased due to 6-MP incubation. From all carbohydrate metabolism related proteins, the glycolysis/gluconeogenesis pathway seemed to be most affected. Related to this pathway, phosphoenolpyruvate carboxykinase has been annotated to different identified bacterial proteins and was decreased after incubation with 6-MP. This enzyme catalyzes Oxaloacetate into Phosphoenolpyruvate [52], which can either be converted into Pyruvate for energy production, or into Glycerate-2P for glucose production and storage [53]. In the same pathway, 2-oxoglutarate/2-oxoacid ferredoxin oxidoreductase subunit alpha was decreased after 6-MP incubation, which can convert Pyruvate into Acetyl-CoA, that is a substrate for short chain fatty acid (SCFA) production, including propionate and butyrate [36]. A decrease in bacterial Acetyl-CoA production may lead to a decrease in SCFA production. In the present study, downregulation of 2-oxoglutarate/2-oxoacid ferredoxin oxidoreductase subunit alpha was specifically annotated to the order of Bacteroidales, including *B. fragilis*, which produces propionate [54]. In contrast, butyrate-producing Firmicutes [54] were not downregulated in this pathway. Consequently, 6-MP intake may increase the relative concentration of luminal butyrate, which can have an effect on the patient’s intestinal physiology and immune response [54]. However, to investigate whether the downregulation of 2-oxoglutarate/2-oxoacid ferredoxin oxidoreductase subunit alpha and phosphoenolpyruvate carboxykinase leads to less propionate production, SCFA concentrations need to be measured in the ex vivo incubations. In case these measurements can confirm a shift in SCFA composition due to 6-MP, SCFA composition needs to be examined in patients before and during 6-MP therapy.

Another group of proteins that were altered in expression levels due to FM incubation with 6-MP includes proteins related to bacterial protein synthesis, including ribosomal proteins. Several different proteins of the aforementioned group were decreased, mainly in Firmicutes and Bacteroidetes, while other proteins were increased, mainly in Proteobacteria.

These findings suggest that RNA translation activity may be suppressed in Bacteroides and Firmicutes but enhanced in Proteobacteria in response to 6-MP. This pattern could reflect differential interactions between 6-MP–derived 6-thioguanine nucleotides and bacterial GTPases, which play key roles in translation [45]. Future mechanistic studies should investigate whether such GTPase-mediated effects underlie phylum-specific differences in translational activity.

In conclusion, protein expression is altered differently among bacterial taxa in response to 6-MP. Consequently, this may lead to taxon-dependent alterations in metabolite or protein production and excretion. Further research should elucidate the subsequent effects on interactions with the host.

Bacterial metaproteomics of commensal ecosystems is an innovative research field, which harbors promising and potentially clinically relevant outcomes. To fully harness this potential, microbial proteome databases need to be expanded to include a broader range of commensal microbes since they are yet mainly focused on pathogens and contain limited protein and pathway annotations for commensals [55]. In our study, less than two-thirds of the significantly increased or decreased proteins could be annotated using KEGG orthology within GhostKOALA [36]. Therefore, additional interesting outcomes may remain undiscovered. Further, the pathway annotation, while informative, should be interpreted with careful consideration since the GhostKOALA tool occasionally annotated human pathways to the identified microbial proteins, despite the query being specified for prokaryotes. This underscores the importance of continued refinement in annotation algorithms to ensure more accurate pathway assignments. Furthermore, taxon annotation using Unipept [37] provided valuable insights; however, some limitations remain. Unipept generates tryptic peptides from the submitted proteins [37], which, in this study, were annotated proteins based on peptide matches that were identified by Proteome Discoverer. This additional processing step might introduce deviations from the original proteins, highlighting a subject of improvement in future analysis tools. While Unipept offered useful taxonomic classifications, a few annotations seem unreliable since they represent pathogens, such as *Vibrio* and *Legionella*, as well as to the marine species *Leucothrix*. These genera are very unlikely to be present in fecal samples of the study populations and were not identified by our bacterial community profiling. These questionable annotations observed in our dataset illustrate the need for improved annotation tools that incorporate habitat-specific taxonomic filtering to enhance reliability.

Although the opportunities for microbial metaproteomic analyses need to be further optimized, our study clearly demonstrates the added value of microbial metaproteomic when compared to bacterial community profiling by unraveling functional microbial responses to common drugs. Furthermore, the rapid workflow and high-throughput expansion capacities offer promising possibilities to apply this tool in clinical drug screening protocols. Thereby, treatment choice may be co-facilitated taking into account microbial drug metabolism and subsequent harmful or complementary effects on intestinal inflammation.

To further increase the knowledge on the effect of drugs on microbiome–host interactions in intestinal inflammation, larger studies are needed which allow the analysis of individual patients and patient subgroups. The combination with clinical multi-omics studies may further pave the way for clinical translation and implementation [12].

## Conclusions

In conclusion, although the CD drugs 6-MP and tofacitinib may not significantly change the microbial composition of intestinal FM incubations, they are able to significantly alter microbial protein expression within 24 h. Thereby, the communication with the host may be altered with subsequent beneficial or harmful effects on intestinal healing. The clinical relevance of drug-induced microbial metaproteomic alterations needs to be further investigated.

## Supplementary Information

Additional file 1. Questionnaire on patient characteristics. The original questionnaire in Dutch language was used to obtain patient characteristics from the fecal sample donors. Questions cover factors that potentially influence the intestinal microbiota, such as body weight, diet, and drug use.

Additional file 2. Table S1: Significantly increased microbial proteins upon 6-MP incubation. This table lists all identified microbial proteins that were increased after FM incubation with 6-MP and shows their respective KEGG orthology as well as pathway annotations.

Additional file 3. Table S2: Significantly decreased microbial proteins upon 6-MP incubation. This table lists all identified microbial proteins that were decreased after FM incubation with 6-MP and shows their respective KEGG orthology as well as pathway annotations.

## Data Availability

The 16S rRNA amplicon sequencing dataset, which is generated during the study is available in the European Nucleotide Archive with accession number PRJEB79375 [56]. The mass spectrometry proteomics data have been deposited to the ProteomeXchange Consortium via the PRIDE [57] partner repository with the dataset accession PXD054714 and 10.6019/PXD054714[58].

## Abbreviations

CD: Crohn’s disease
6-MP: 6-Mercaptopurine
FM: Fecal microbiota
IBD–SL: Inflammatory Bowel Disease South Limburg
DMSO: Dimethylsulfoxide
LC-MS: Liquid chromatography–mass spectrometry
DTT: Dithiothreitol
ABC: Ammoniumbicarbonate
ASV: Amplicon sequence variant
PCoA: Principal coordinate analysis
PERMANOVA: Permutational multivariate analysis of variance
LinDA: Linear Models for Differential Abundance Analysis
FDR: False discovery rate
PPI: Proton pump inhibitor
BMI: Body mass index
HBI: Harvey Bredshaw Index
BSS: Bristol Stool Scale
JAK: Janus kinase
SCFA: Short-chain fatty acid
