# Supplementary material for: 6-mercaptopurine and tofacitinib alter microbial protein expression but not composition in fecal microbiota incubations from Crohn’s disease patients
Source: BMC Biol. 2026 Mar 14;24:101. doi: 10.1186/s12915-026-02569-9 (PMC13101326; doi:10.1186/s12915-026-02569-9)
Supplement: Supplementary file 1 — Additional file 1. Questionnaire on patient characteristics. The original questionnaire in Dutch language was used to obtain patient characteristics from the fecal sample donors. Questions cover factors that potentially influence the intestinal microbiota, such as body weight, diet, and drug use. [file 12915_2026_2569_MOESM1_ESM.pdf]

Vragenlijst Farmacomicrobiota (versie 2, dd 04-12-2019)

Volgnummer: ____ **Datum:** ____

Tijd opvang ontlasting: ____

Lengte: ____ m **Leeftijd:** ____

Gewicht: ____ kg **Geslacht:** ____

BMI: ____ kg/m^2

Roken: huidig <6 mnd gestopt >6 mnd gestopt nooit gerookt

Gebruik PPI*: ja / nee
(afgelopen week)
Welke: ____

Startdatum: ____ **Stopdatum:** ____

Medicatiegebruik (afgelopen week t/m vandaag vooraf aan opvang ontlasting)
Welke: ____

Medicatieveranderingen: ja / nee
(afgelopen week)
Welke: ____

Probioticagebruik: ja / nee
(afgelopen 7 dagen incl. vandaag)
Welke: ____

Alternatief dieet: ja/nee
(bv. Vegetarisch/veganistisch, vezelrijk/-arm, volgens religieuze voorschriften, macrobiotisch, antroposofisch, lactosevrij, glutenvrij, low-FODMAP etc.)
Zo ja, welk: ____

Zwangerschap: ja / nee

Dit formulier heeft betrekking op de dag van verzameling faeces monster.
*PPI= proton pump inhibitor = "maagzuur remmer" (bv. omeprazol, pantoprazol)
